# Risk Factors for Late HIV Presentation in Patients Treated at a Single Belgian Reference Centre from 2018 to 2022

**DOI:** 10.3390/idr16020019

**Published:** 2024-03-14

**Authors:** Damien Scaia, Karine Fombellida, Nathalie Maes, Majdouline El Moussaoui, Gilles Darcis

**Affiliations:** 1 Public Health Science Department, University of Liège, 4000 Liège, Belgium; 2 Infectious Diseases Department, Liège University Hospital, 4000 Liège, Belgium; 3 Biostatistics and Research Method Center, Liège University Hospital, 4000 Liège, Belgium

**Keywords:** HIV, late presentation, AIDS, advanced HIV, risk factors, delayed HIV diagnosis

## Abstract

A late HIV diagnosis is associated with increased mortality and morbidity, increased healthcare costs and increased onward viral transmission. In this regard, we retrospectively analysed the characteristics of patients who presented for care at our centre from January 2018 to December 2022 to assess the proportion of patients and factors associated with late HIV presentation. We collected data from the Liège University Hospital database, and we used binary logistic regression models to analyse the impact of individuals’ characteristics on late presentation. Among 167 participants, 38.3% were late presenters (LPs) (presenting for care with a CD4^+^ T-cell count < 350 cells/mm^3^ or after an AIDS-defining event), and 21.6% were late presenters with advanced disease (LPs-AD) (presenting for care with a CD4^+^ T-cell count < 200 cells/mm^3^ or after an AIDS-defining event). The risk of being an LPs-AD was increased in older individuals (OR on log-transformed age: 7.5) and individuals of sub-Saharan African origin compared to individuals of Belgian or other origin (ORs of 0.30 and 0.25, respectively). The results of this study suggest that broadening the focus beyond the previously common risk groups is essential to prevent late diagnosis.

## 1. Introduction

With the introduction of antiretroviral therapy (ART) in 1996, HIV-associated morbidity and mortality have significantly decreased. Today, the life expectancy of people living with HIV (PLWH) is close to that of the general population provided that they receive timely diagnosis and start treatment when their CD4 counts are ≥350 cells/μL [1]. However, despite the considerable advances achieved in the field, PLWH are often diagnosed at a late disease stage, defined as having a CD4^+^ T-cell count less than 350 cells/μL or presenting with an AIDS-defining event, regardless of the CD4^+^ T-cell count [2,3]. Late HIV presentation is a persistent issue in the global fight against HIV because it is associated with increased mortality and morbidity [4,5,6], increased medical care costs [7,8,9,10] and increased sexual and vertical transmission due to late ART initiation [6,10,11]. Early initiation of ART, especially when the CD4^+^ T-cell count is greater than 350/mm^3^, offers many benefits, including improved immunological recovery and subsequent reductions in both severe AIDS-related and severe non-AIDS-related events, as well as limited establishment of HIV reservoirs [12,13]. Therefore, it is critical to identify factors associated with late presentation for HIV care to improve the effectiveness of HIV testing strategies, with a focus on susceptible and possibly neglected subgroups.

Nonetheless, it is important to assess each region’s specific population and epidemiological context to determine the most appropriate response against HIV. In a previously published study, we analysed the characteristics of patients who presented for care at our centre from 2006 to 2017. Forty-four percent of the patients were late presenters [14]. Here, we aimed to determine the proportion and predictors of late presentation (LP) and late presentation with advanced HIV disease (LP-AD) in recent years.

## 2. Materials and Methods

In this retrospective cohort study, we included HIV-1-infected individuals aged at least 16 years who presented for clinical care at Liège University Hospital (Belgium) for the first time between 1 January 2018 and 31 July 2022. Patients who had already received care for HIV infection at another clinical centre as well as patients who were treated for more than six months before the first clinic visit were excluded from the study.

According to a consensus definition of late presenters, “the term ‘presentation for care’ means attendance at a health care facility that is able to monitor progression of HIV infection and initiate appropriate medical care, including ART, as appropriate” [15]. Consequently, people were also included if they had already been diagnosed but had not received care for their infection.

Patients were classified according to their CD4^+^ T-cell count at ART initiation and history of AIDS-defining events within the six months following the first visit [15]. Late HIV presenters (LPs) were defined as individuals with a CD4^+^ T-cell count < 350 cells/mm^3^ at presentation for care or who experienced an AIDS-defining event in the six months following the first visit. LPs were further stratified as late presenters with advanced HIV disease (LPs-AD) if the CD4^+^ T-cell count was <200 cells/mm^3^ at presentation for care or if an AIDS-defining event occurred (regardless of the CD4^+^ T-cell count) in the six months following their first visit. Seroconverters (individuals with an HIV-negative test or a clinical history of primary infection over the past 12 months) with a CD4^+^ T-cell count <350 cells/mm^3^ were classified as non-LPs. By definition, such people were diagnosed soon after HIV infection and would not have been reached by interventions that promoted earlier targeted screening. Patients infected with HIV-2 were excluded from the analyses because the kinetics of the decrease in the CD4^+^ T-cell count differed in these patients.

We collected the baseline characteristics of the PLWH from the electronic medical registry. These data included demographic and clinical characteristics, including age, sex, ethnicity, country of origin, mode of acquisition, information regarding AIDS-defining illnesses and the first CD4^+^ T-cell count and year of first presentation for care. Finally, we also recorded the screening context, categorised as voluntary screening, medical condition screening, refugee (defined as a person fleeing armed conflict or persecution and asking for or benefiting from assistance by the state, the Red Cross or other organisations) screening, incidental screening (for instance, before a blood donation), pregnancy screening, screening due to having an HIV+ partner or other/unknown reasons. These variables were chosen because they are commonly studied in the literature [14,16,17,18], which allowed for comparisons with past studies and especially with our previous study [14].

### Statistical Analysis

The normality of the distribution of each quantitative variable was investigated using means and medians, Shapiro–Wilk tests, histograms, quantile–quantile plots (Q–Q plots) and box plots. As the quantitative variables did not have a normal distribution, they are summarised as medians, interquartile ranges (IQRs) and extreme values. Categorical variables are summarised with frequency tables. Development over time of late presentation (LP and LP-AD) as well as the impact of individuals’ characteristics on late presentation were analysed using binary logistic regression models. Univariate logistic models were constructed, and variables with *p* values lower than 0.10 were selected for multivariable logistic models. Collinearity between origin and ethnicity led us to include only origin in the multivariate models. For univariate models, adjusted *p* values (Holm–Bonferroni method) are also presented. To identify potential factors associated with late presentation, participants were further divided into 3 subgroups according to their CD4+ T-cell count and AIDS-defining event status.

Analyses were carried out on the maximum amount of data available, and no missing values were replaced. The results were considered significant at the 10% alpha level (*p* < 0.10). Data analysis was performed using SAS (version 9.4 (SAS Institute Inc., Cary, NC, USA) for Windows) and R (version 4.2.1 (The R Foundation for Statistical Computing, Vienna, Austria) for Windows).

Approval for the study protocol was obtained from the local ethics review committee (Comité d’Ethique Hospitalo-Facultaire Universitaire de Liège, reference number 2023-39). Individual consent was waived due to the retrospective nature of the study and the anonymisation of the data.

## 3. Results

### 3.1. Study Cohort Characteristics

**Figure 1:**
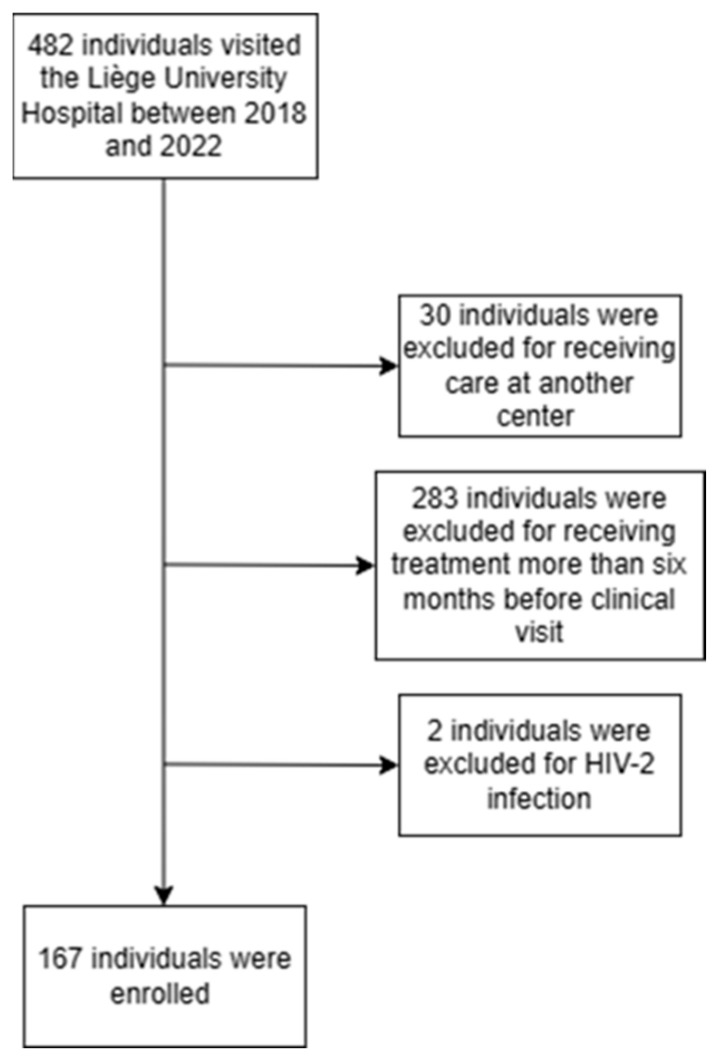
Study flowchart.

Of the 482 patients who presented for care at the University Hospital of Liège between 1 January 2018 and 31 December 2022, 30 and 283 individuals were excluded because they had been previously followed at another centre or treated for more than six months, respectively. Additionally, two patients infected with HIV-2 were also excluded. Overall, 167 participants were eligible for the analysis (Figure 1). The characteristics of the 167 participants who first presented for care are shown in Table 1. Most individuals were male (70.7%), and African was the most common ethnicity (45.5%). The median age was 37 years (range, 18–79). First, the CD4^+^ T-cell count was less than 350 cells/mm^3^ for 34 participants (20.4%) and less than 200 cells/mm^3^ for 36 participants (21.6%). Regarding the route of HIV transmission, HIV was transmitted by a heterosexual partner for 71 PLWH (42.5%) and by a homo- or bisexual partner for 69 PLWH (41.3%). The screening tests were mostly performed during investigations for medical problems (37.7%) or in the refugee population (21.6%). In contrast, screening tests were less frequently performed during pregnancy (1.8%). Eighteen participants (10.8%) had an opportunistic infection at diagnosis and presented for HIV care for the first time after a median time of 3.5 days after their diagnosis. Pneumocystis pneumonia (PCP), neurotoxoplasmosis, HIV-related cachexia, HIV-related encephalopathy, cytomegalovirus retinitis, cytomegalovirus infection other than retinitis, pulmonary and extrapulmonary tuberculosis, progressive multifocal leukoencephalopathy and Kaposi’s sarcoma were among the AIDS-defining conditions presented by the patients.

Table S1 shows the breakdown of care by year, and population characteristics are shown in Table 1. Among the 167 patients, 64 (38.3%) were LPs, and 36 (21.6%) were LPs-AD (Table 2). Among the remaining 103 individuals in the non-LPs group, two were initially considered LPs-AD, and four were considered LPs. However, those six individuals were moved to the non-LP group since they had a negative HIV test result less than one year before their diagnosis.

Among the sample of 167 individuals, the main group was Belgian men who have sex with men (MSM) (n = 34; 20.4%), but they did not account for the majority of male individuals (28.8%) (Tables S2 and S3). However, they represented close to the majority of the homo/bisexual group (49.3%) (Table S4). Thirty-three individuals (19.8%) were heterosexual women from SSA, representing close to the majority of the heterosexual transmission group (46.5%) (Tables S2 and S5).

Table 3 shows the characteristics of the population in the LP and LP-AD groups. As the “pregnancy” modality for the variable “context of screening” included few people compared to the other modalities, it was combined with the modality “unknown” to avoid quasi-complete separation in the logistic regression.

### 3.2. Factors Associated with Late Presentation and Late Presentation with Advanced Disease

The factors associated with LP and LP-AD are displayed in Table 4 and Table 5, respectively. Sex, ethnicity, origin and mode of acquisition were the factors associated with late presentation (Table 4), whereas age, ethnicity and origin were the factors associated with LP-AD (Table 5).

The multivariate models (Table 6 and Table 7) included the variables that were statistically significant in the univariate models (Table 4 and Table 5). Ethnicity was not included in the multivariate models because it was linked to the variable “Origin” (Chi^2^ *p* < 0.0001). According to the multivariate model of “LP vs. Non-LP” (Table 6), no variable remained significantly associated with late presentation. According to the multivariate model of “LP-AD vs. Non-LP” (Table 7), both age and origin were significantly associated with late presentation of advanced HIV disease.

Older age and a sub-Saharan African origin significantly increased the risk of LP-AD (*p* = 0.010). A person of Belgian origin was less likely to have a late presentation with advanced HIV disease than a person of sub-Saharan African origin. A person of another origin also had a lower probability of late presentation with advanced HIV disease than a person of sub-Saharan African origin. The LP and LP-AD rates varied from year to year but did not change significantly over time (LP: *p* = 0.80, LP-AD: *p* = 0.70) (Tables S1, S4 and S5 and Figure S1).

## 4. Discussion

Compared to our previous study [14], many changes were observed between the time periods 2006–2017 and 2018–2022. First, the population was older (median age of 34.0 years (IQR 27.0–42.0) vs. 37.0 years (IQR 29.0–45.5)) in 2018–2022. Although heterosexual transmission still remained the most common mode of acquisition, it decreased from 61.0% in 2006–2017 to 42.5% in 2018–2022. In contrast to the 2006–2017 period, although Belgian MSM and heterosexual women from SSA remained the most represented groups, they did not account for the majority of individuals in the cohort. This means that other population profiles are becoming more prevalent, resulting in a diversified epidemic, which is corroborated by the literature [19].

We found that older patients and patients from sub-Saharan Africa were at risk of late presentation with advanced HIV disease. This could be explained by patients’ fear of the perceived consequences of a positive test result, notably the fear of stigma and discrimination [20,21,22]. Past adverse experiences such as loss of acquaintances during the early days of the HIV epidemic may also explain late testing and late presentation, especially among older patients [22].

Our results differ from the LP risk factors reported in our previous study, which highlighted age, male sex, non-Belgian origin and heterosexual transmission as risk factors for late presentation and age, non-Belgian origin, heterosexual transmission and testing for medical conditions as risk factors for late presentation with advanced HIV disease [14]. The discrepancies between the two studies can be explained by the small sample size of the present study, which included 167 individuals compared to the 604 individuals included in the previous study. This lack of statistical power would also explain the wide confidence intervals observed for some variables, such as age (Table 4 and Table 5). The large number of individuals excluded due to receiving treatment more than six months before their clinical visit can be explained by Belgium’s link to sub-Saharan Africa, notably the Democratic Republic of the Congo (past colony), which results in many migrants from this region.

Other studies performed during a similar timeframe (2017 to 2020, 2019 to 2020 and 2015 to 2020) also revealed that male and older patients are at greater risk of late HIV presentation [23,24,25]. Additionally, other LP risk factors, including bisexual transmission, heterosexual transmission, being married, SSA origin, tuberculosis or hepatitis C virus coinfection, lower socioeconomic status and the fear of stigma, have been reported in various studies [25,26,27,28,29,30,31,32,33]. The discrepancies observed among those studies highlight the importance of identifying populations at risk of late presentation and developing specific guidelines according to the region.

Notably, this study aimed to determine the proportions and predictors of LP and LP-AD between 2018 and 2022, a period marked by the COVID-19 pandemic. This pandemic may have influenced our findings, as there was a decrease in new HIV diagnoses, in the follow-up of PLWH and in comorbidity screening during the first wave of the pandemic in Belgium [34,35]. This could be explained by several factors, such as the restrictions on medical appointments, the closing of several screening centres and the misinterpretation of symptoms of HIV infection as symptoms of COVID-19 infection.

The possible bias and limitations of this study should be noted. First, limiting the study population to individuals presenting for care at Liège University Hospital may have influenced the results, as profiles may be limited, resulting in selection bias. A greater number of participants is needed to draw more solid conclusions about the at-risk population. Conversely, this study’s strength lies in the fact that the chosen healthcare facility includes the “AIDS reference centre of Liège”, which provides individuals and their families with multidisciplinary care and is “hidden” within the facility (“AIDS reference centre” is not written anywhere) [36]. This may have resulted in widening the scope of individuals presenting at the facility and may have attenuated the selection bias.

Since additional risk factors for late presentation could have been missed, it would be interesting to extend this study to other testing centres in Liège to better investigate the potential impact of the real diversity of the population. It would also be interesting to complete a qualitative study among late presenters to assess the impact of the COVID-19 pandemic on late presentations.

Since most screenings were performed due to a medical problem, voluntary screening should be promoted through information campaigns. Although these information campaigns should focus on high-prevalence groups, they should also target other groups, as the variety of profiles of late presenters seems to be increasing.

## 5. Conclusions

We investigated the evolution of the proportion of individuals with LP and factors associated with late HIV presentation among patients treated at the University Hospital of Liège in Belgium between 2018 and 2022. The risk of late presentation with advanced HIV disease was increased in older individuals and individuals of sub-Saharan African origin. It is therefore particularly important to develop information and screening strategies targeting these risk groups. Although the LP and LP-AD rates varied from year to year, they did not decrease significantly over time, which suggests the need for additional efforts to promote earlier diagnosis. Our results highlight the complexity of late HIV diagnosis and presentation for care and will hopefully help in controlling the AIDS epidemic.

## Figures and Tables

**Table 1:** Population characteristics (n = 167).

	N (%)	Median (IQR)	Extremes
Age (years)	167	37.0 (29.0–45.5)	18.0–79.0
Sex			
Male	118 (70.7)		
Female	49 (29.3)		
Ethnicity			
Caucasian	68 (40.7)		
African	76 (45.5)		
Others	23 (13.8)		
Origin			
Belgium	59 (35.3)		
Sub-Saharan Africa	75 (44.9)		
Others	33 (19.8)		
Mode of acquisition			
Heterosexual transmission	71 (42.5)		
Homo/Bisexual transmission	69 (41.3)		
Others/Unknown	27 (16.2)		
First CD4 T-cell count			
Number/mm^3^	167	414.0 (245.0–621.50)	1.0–1322.0
<200 cells/mm^3^	36 (21.6)		
200–349 cells/mm^3^	34 (20.4)		
≥350 cells/mm^3^	97 (58.1)		
Context of screening			
Incidental screening	22 (13.2)		
Voluntary screening	24 (14.4)		
HIV-positive partner	11 (6.6)		
Medical problem	63 (37.7)		
Pregnancy	3 (1.8)		
Refugee	36 (21.6)		
Unknown	8 (4.8)		
Number of days between opportunistic infection and first presentation for care	18 (10.8)	−3.5 (−28.8–9.8)	−417.0–1550.0

**Table 2:** Late presentation for care (n = 167).

	N (%)
Non-LPs	103 (61.7)
LPs ^1^	64 (38.3)
LPs-AD ^2,^*	36 (21.6)

^1^ CD4 T-cell count < 350 cells/mm^3^ or an AIDS-defining event (at any CD4 count) in the six months following the first visit. ^2^ CD4 T-cell count < 200 cells/mm^3^ or an AIDS-defining event (at any CD4 count) in the six months following the first visit. * Subgroup of LPs.

**Table 3:** Percentages of non-late presenters (Non-LPs), late presenters (LPs) and late presenters with advanced HIV disease (LPs-AD) as a function of the population characteristics (n = 167).

	N	%Non-LPs	%LPs	%LPs-AD *
Age				
<30 years	47	70.2%	29.8%	10.6%
30–50 years	95	55.8%	44.2%	26.3%
>50 years	25	68.0%	32.0%	24.0%
Sex				
Male	118	66.9%	33.1%	20.3%
Female	49	49.0%	51.0%	24.5%
Ethnicity				
Caucasian	68	76.5%	23.5%	16.2%
African	76	47.4%	52.6%	27.6%
Others	23	65.2%	34.8%	17.4%
Origin				
Belgium	59	71.2%	28.8%	18.6%
Sub-Saharan Africa	75	48.0%	52.0%	28.0%
Others	33	75.8%	24.2%	12.1%
Mode of acquisition				
Heterosexual transmission	71	52.1%	47.9%	26.8%
Homo/Bisexual transmission	69	73.9%	26.1%	15.9%
Others/Unknown	27	55.6%	44.4%	22.2%
Context of screening				
Incidental screening	22	68.2%	31.8%	9.1%
Voluntary screening	24	83.3%	16.7%	12.5%
HIV-positive partner	11	54.5%	45.5%	27.3%
Medical problem	63	60.3%	39.7%	28.6%
Pregnancy	3	33.3%	66.7%	0.0%
Refugee	36	50.0%	50.0%	22.2%
Unknown	8	62.5%	37.5%	25.0%
Year of presentation for care				
2018	39	61.5%	38.5%	28.2%
2019	41	53.7%	46.3%	24.4%
2020	31	64.5%	35.5%	16.1%
2021	27	66.7%	33.3%	18.5%
2022	29	65.5%	34.5%	17.7%

* Subgroup of LPs.

**Table 4:** Factors associated with LP—univariate logistic regression.

	LP Group vs. Non-LP Group (N = 167)			
	Coefficient ± SE	Odds Ratio (95% CI)	*p* Value	Adjusted *p* Value
Age *	0.75 ± 0.51	2.11 (0.79–5.84)	0.14	0.42
Sex (Ref = Male)				
Female	0.75 ± 0.35	2.11 (1.07–4.18)	0.031	0.12
Ethnicity (Ref = African)			0.0013	0.0091
Caucasian	−1.28 ± 0.37	0.28 (0.13–0.56)	<0.001	
Others	−0.73 ± 0.49	0.48 (0.17–1.24)	0.14	
Origin (Ref = Sub-Saharan Africa)			0.0040	0.024
Belgium	−0.98 ± 0.37	0.37 (0.18–0.76)	0.0076	
Others	−1.22 ± 0.47	0.30 (0.11–0.71)	0.0091	
Mode of acquisition (Ref = Heterosexual transmission)			0.021	0.10
Homo/Bisexual transmission	−0.96 ± 0.36	0.38 (0.19–0.77)	0.008	
Others/Unknown	−0.14 ± 0.45	0.87 (0.35–2.12)	0.76	
Context of screening (Ref = Voluntary screening)			0.21	0.42
Incidental screening	0.85 ± 0.71	2.33 (0.58–9.45)	0.24	
HIV-positive partner	1.43 ± 0.82	4.17 (0.84–20.64)	0.081	
Medical problem	1.19 ± 0.61	3.29 (1.00–10.77)	0.049	
Refugee	1.61 ± 0.64	5.00 (1.42–17.57)	0.012	
Others/Unknown	1.43 ± 0.82	4.16 (0.84–20.64)	0.081	
Year of presentation for care (Ref = 2018)			0.80	0.80
2019	0.32 ± 0.45	1.38 (0.57–3.40)	0.48	
2020	−0.13 ± 0.50	0.88 (0.33–2.34)	0.80	
2021	−0.22 ± 0.52	0.80 (0.28–2.22)	0.67	
2022	−0.17 ± 0.51	0.84 (0.30–2.28)	0.74	

* Normalised variable using log transformation.

**Table 5:** Factors associated with LP-AD—univariate logistic regression.

	LP-AD Group vs. Non-LP Group (N = 139)			
	Coefficient ± SE	Odds Ratio (95% CI)	*p* Value	Adjusted *p* Value
Age *	1.53 ± 0.64	4.63 (1.36–17.26)	0.017	0.12
Sex (Ref = Male)				
Female	0.50 ± 0.42	1.65 (0.70–3.75)	0.24	0.72
Ethnicity (Ref = African)			0.048	0.24
Caucasian	−1.01 ± 0.43	0.36 (0.15–0.83)	0.019	
Others	−0.78 ± 0.63	0.46 (0.12–1.46)	0.21	
Origin (Ref = Sub-Saharan Africa)			0.037	0.22
Belgium	−0.80 ± 0.44	0.45 (0.19–1.04)	0.066	
Others	−1.29 ± 0.60	0.27 (0.073–0.83)	0.032	
Mode of acquisition (Ref = Heterosexual transmission)			0.12	0.48
Homo/Bisexual transmission	−0.87 ± 0.44	0.42 (0.17–0.97)	0.047	
Others/Unknown	−0.25 ± 0.56	0.78 (0.24–2.26)	0.66	
Context of screening (Ref = Voluntary screening)			0.31	0.62
Incidental screening	−0.12 ± 0.97	0.89 (0.11–6.02)	0.90	
HIV-positive partner	1.20 ± 0.94	3.33 (0.50–22.72)	0.20	
Medical problem	1.15 ± 0.68	3.16 (0.93–14.63)	0.09	
Refugee	1.09 ± 0.75	2.96 (0.73–15.14)	0.15	
Others/Unknown	0.80 ± 1.02	2.22 (0.25–16.80)	0.44	
Year of presentation for care (Ref = 2018)			0.73	0.73
2019	−0.010 ± 0.53	0.99 (0.35–2.80)	0.99	
2020	−0.61 ± 0.62	0.55 (0.15–1.77)	0.33	
2021	−0.50 ± 0.62	0.61 (0.17–1.99)	0.42	
2022	−0.55 ± 0.62	0.57 (0.16–1.87)	0.37	

* Normalised variable using log transformation.

**Table 6:** Factors associated with LP—multivariate logistic regression (n = 167).

	LP vs. Non-LP		
	Coefficient ± SE	Odds Ratio (95% CI)	*p* Value
Sex (Ref = Male)			
Female	0.043 ± 0.44	1.044 (0.43–2.50)	0.92
Origin (Ref = Sub-Saharan Africa)			0.084
Belgium	−0.77 ± 0.41	0.46 (0.20–1.03)	0.061
Others	−0.97 ± 0.52	0.38 (0.13–1.03)	0.064
Mode of acquisition (Ref = Heterosexual transmission)			0.42
Homo/Bisexual transmission	−0.51 ± 0.46	0.60 (0.24–1.48)	0.27
Others/Unknown	0.081 ± 0.49	1.084 (0.41–2.84)	0.87

**Table 7:** Factors associated with LP-AD—multivariate logistic regression (n = 139).

	LP-AD vs. Non-LP		
	Coefficient ± SE	Odds Ratio (95% CI)	*p* Value
Age *	2.01 ± 0.72	7.48 (1.92–33.35)	0.010
Origin (Ref = Sub-Saharan Africa)			0.010
Belgium	−1.20 ± 0.48	0.30 (0.11–0.75)	0.013
Others	−1.40 ± 0.63	0.25 (0.06–0.77)	0.025

* Normalised variable using log transformation.

## Data Availability

The data presented in this study are available from the corresponding authors upon request.

## Supplementary Materials

The following supporting information can be downloaded at https://www.mdpi.com/article/10.3390/idr16020019/s1: Table S1: Presentation for care: evolution over time; Table S2: Groups in the cohort; Table S3: Mode of acquisition among men; Table S4: Homo/Bisexual transmission by sex and origin; Table S5: Heterosexual transmission by sex and origin; Figure S1: Evolution of LP and LP-AD rates over time.
