# Supplementary material for: Risk Factors for Late HIV Presentation in Patients Treated at a Single Belgian Reference Centre from 2018 to 2022
Source: Infect Dis Rep. 2024 Mar 14;16(2):239–48. doi: 10.3390/idr16020019 (PMC10961754; doi:10.3390/idr16020019)
Supplement: Supplementary file 1 [file idr-16-00019-s001.zip › idr-2786098-supplementary.pdf]

Risk factors of HIV late presentation in a single Belgian reference center

Authors

Damien Scaia [1,*], Karine Fombellida [2], Nathalie Maes [3], Majdouline El Moussaoui [2], and Gilles Darcis [2,*]

Affilitation

1 Public Health Science Department, University of Liège, 4000 Liège, Belgium

2 Infectious Diseases Department, Liège University Hospital, 4000 Liège, Belgium

3 Biostatistics and Research Method Center, Liège University Hospital, 4000 Liège, Belgium

Supplementary information

Table S1. Presentation for care: evolution over time (n=167)

Year of presentation for care	Number of individuals	N (%) LP [1]	N (%) LP-AD [2]
2018	39	15 (38.5)	11 (28.2)
2019	41	19 (46.3)	10 (24.4)
2020	31	11 (35.5)	5 (16.1)
2021	27	9 (33.3)	5 (18.5)
2022	29	10 (34.5)	5 (17.2)
p-value [3]		0.80	0.73

[1] CD4 T cells <350/mm³ or an AIDS-defining event (at any CD4) in the six months following first visit
[2] CD4 T cells <200/mm³ or an AIDS-defining event (at any CD4) in the six months following first visit
[3] Logistic regression

Table S2. Groups in the cohort (n=167)

	Origin		
	Belgium	Sub-Saharan Africa	Others
Heterosexual men	12 (7.19%)	16 (9.58%)	2 (1.20%)
Heterosexual women	7 (4.19%)	33 (19.76%)	1 (0.60%)
Homo/Bisexual men	34 (20.36%)	13 (7.78%)	22 (13.17%)
Homo/Bisexual women	0 (0%)	0 (0%)	0 (0%)
Others	6 (3.59%)	13 (7.78%)	8 (4.79%)

Table S3. Mode of acquisition among men (n=118)

	Mode of acquisition		
	Heterosexual transmission	Homo/Bisexual transmission	Others/Unknown
Origin			
Belgium	12 (10.17%)	34 (28.81%)	6 (5.09%)
Sub-Saharan Africa	16 (13.56%)	13 (11.02%)	6 (5.09%)
Others	2 (1.70%)	22 (18.64%)	7 (5.93%)

Table S4. Homo/Bisexual transmission by gender and origin (n=69)

	Origin		
	Belgium	Sub-Saharan Africa	Others
Gender			
Male	34 (49.28%)	13 (18.84%)	22 (31.88%)
Female	0 (0%)	0 (0%)	0 (0%)

Table S5. Heterosexual transmission by gender and origin (n=71)

	Origin		
	**Belgium**	**Sub-Saharan Africa**	**Others**
Gender			
Male	12 (16.90%)	16 (22.54%)	2 (2.82%)
Female	7 (9.86%)	33 (46.48%)	1 (1.41%)

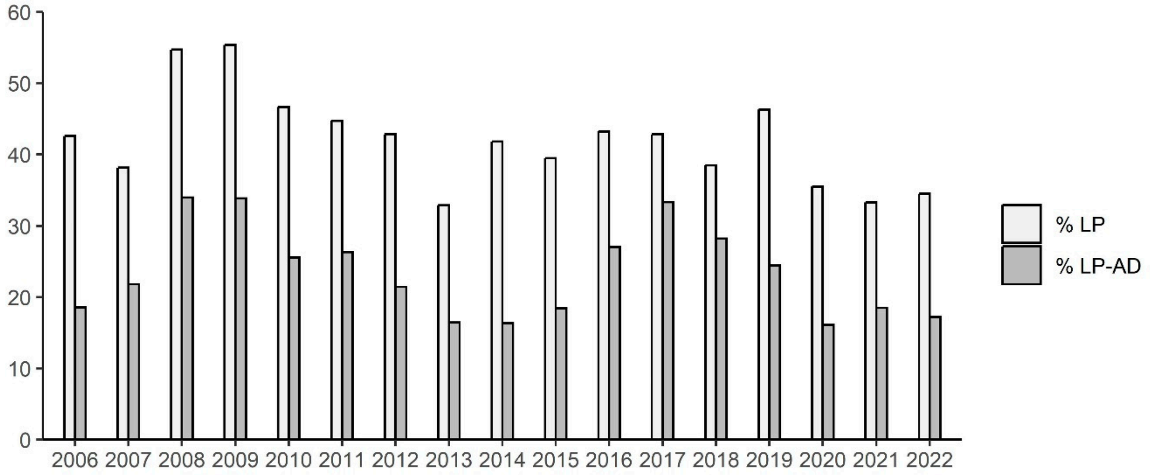

Figure S1. Evolution of LP and LP-AD rates over time
